# Optimizing the Extraction of Bioactive Compounds (Polyphenols, Lipids, and Alpha-Tocopherol) from Almond Okara to Unlock Its Potential as Functional Food

**DOI:** 10.3390/foods13172828

**Published:** 2024-09-05

**Authors:** Mariam Taha, Krasimir Dimitrov, Jennifer Samaillie, Benjamin Caux, Sevser Sahpaz, Nicolas Blanchemain, Caroline West, Céline Rivière

**Affiliations:** 1 BioEcoAgro, Joint Research Unit 1158, University of Lille, INRAE, University of Liège, UPJV, JUNIA, University of Artois, University Littoral Côte d’Opale, ICV—Institut Charles Viollette, 59650 Villeneuve-d’Ascq, Franceceline.riviere@univ-lille.fr (C.R.); 2 ICOA, CNRS UMR 7311, Pôle de Chimie, University of Orleans, Rue de Chartres BP 6759, CEDEX 2, 45067 Orleans, France; 3 INSERM, CHU Lille, U1008—Advanced Drug Delivery Systems, 59000 Lille, France

**Keywords:** almond milk by-product, bioactive compounds, solid-liquid extraction, polyphenols, antioxidant activity, alpha-tocopherol, triglycerides

## Abstract

Almond okara, a by-product of almond milk production, is rich in bioactive components, such as polyphenols, lipids, and alpha-tocopherol, making it a valuable functional food ingredient. This work aimed to investigate its composition while exploring two main aspects: (i) the impact of extraction time, solid-to-solvent ratio, ethanol concentration, and temperature on polyphenol recovery, and (ii) the quantification of okara’s triglycerides (TG) and alpha-tocopherol contents. The polyphenols’ optimal extraction conditions were 90 min, a 1:30 solid-to-solvent ratio (*w*/*v*), 50% ethanol, and 60 °C. These conditions achieved a total polyphenol yield of 523 mg GAE, tannin yield of 340 mg GAE, total flavonoid yield of 548 mg CE, and a total antioxidant capacity of 779 mg AAE per 100 g dry okara. The Peleg model effectively described the extraction kinetics. Additionally, TG levels, quantified by UHE/LPSFC-APCI-MS, in okara were comparable to those in almonds, and alpha-tocopherol levels, quantified by LC-UV, were 14,400 µg/100 g in almonds and 15,600 µg/100 g in okara. These findings highlight the potential of okara as a valuable resource, with a straightforward, scalable, and cost-effective solid-liquid extraction (SLE) method for polyphenols and a supercritical fluid extraction method for TG, for use in the functional food, nutraceutical, and cosmetic industries.

## 1. Introduction

Almond (*Prunus amygdalus* Batsch, syn. *Prunus dulcis* Mill., Rosaceae) milk is among the four most consumed plant-based beverages worldwide with an estimated market size of USD 5.49 billion in 2024 [1]. The widespread belief that it is a high-protein, low-calorie, and low-fat beverage, along with shifting dietary choices, the increasing popularity of veganism, the well-known health advantages of almonds, and its suitability as a substitute for those who are lactose intolerant [2,3], all contribute to its rising popularity.

Almond kernels exhibit a varied phenolic profile, mainly proanthocyanidins, hydrolysable tannins, flavonoids, and phenolic acids, with mean percentiles of 162, 82.1, 61.2, and 5.5 mg/100 g of almond respectively [2,4,5,6,7]. These phytochemicals contribute to various beneficial actions on health, including antioxidant, antimicrobial, and anti-inflammatory activities, as well as intestinal microbiota modulation, promoting gut health and reducing the number of risk factors associated with type 2 diabetes, obesity, and cardiovascular diseases [6,8,9,10,11].

Moreover, almonds are a rich source of lipids, with approximately 50 g of healthy fats per 100 g of almonds, of which there are 40 g of mono- and polyunsaturated fatty acids (MUFAs and PUFAs), and only 4 g of saturated fats [12]. It is well established that almond lipid fractions, mainly MUFAs and PUFAs, ameliorate blood lipid levels. Daily consumption of almonds results in an improvement in the lipid profile, including a reduction in serum levels of total cholesterol (TC), low-density lipoprotein cholesterol (LDL-C), non-high-density lipoprotein (non-HDL-c), and triglycerides (TG), as well as a reduction in body fat and waist-to-hip ratio [13].

Additionally, almonds contain other fat-soluble bioactive compounds, the most important of which is vitamin E or tocopherol, primarily α-, followed by γ-, δ-, and β-tocopherol analogs [14,15]. This antioxidant vitamin plays an important role in protecting PUFAs against peroxidation [9,12,16] and is highly effective in preventing the complications of various diseases. It also has anti-inflammatory processes, inhibits platelet aggregation, and has immune system-strengthening activity [17].

A substantial amount of solid residue, also known as cake, bagasse, or okara, is generated during almond milk production. Despite its high nutritional potential owing to the richness of almonds in phytochemicals, such as phenolic compounds, lipids, proteins, and vitamin E, this by-product remains under-researched, undervalued, and underutilized. Furthermore, the cultivation of almonds presents a significant environmental impact due to the high-water requirements of almond orchards. Therefore, the valorization of the almond milk by-product offers a double advantage. Firstly, it allows the inclusion of bioactive compounds from almond okara in the development of functional food and active ingredients for the cosmetic and pharmaceutical industries. Secondly, it helps to reduce the environmental impact of almond cultivation by promoting a circular economy, thus enhancing the sustainability of the almond milk industry [18,19,20]. The current literature does little to document the chemical composition of this almond by-product, nor the optimization of extraction conditions for some of these constituents such as polyphenols, lipids, and vitamin E. To date, most research has focused on the protein and fiber composition of almond okara and its antioxidant activity [21,22,23].

The present work focused on the less-emphasized polyphenols extraction of almond okara. The efficiency of polyphenol extraction is known to depend on several critical parameters, such as extraction solvent, pH, time, temperature, the solid-to-solvent ratio, etc. [24]. Currently, solid–liquid extraction (SLE) with water and ethanol as extraction solvents, widely used for polyphenol extraction from plant matrices, is the first choice for polyphenol extraction from okara. It offers several advantages over ultrasound-assisted extraction (UAE) and microwave-assisted extraction (MAE), such as ease of use, lower initial investment, ideal scalability, controllable extraction conditions, cost-effectiveness, etc. The solvent of SLE, ethanol in particular, is favored for its high efficacy in polyphenol extraction, non-toxicity, environmental acceptability, and lack of regulatory issues in the food and nutraceutical industries [25].

While SLE is a widely used and versatile extraction method, its disadvantages, such as longer extraction times, lower efficiency, and higher solvent consumption, highlight the need for careful consideration of the optimization of the method concerning the specific requirements of the extraction process.

The present work aimed to maximize yields and ensure the efficiency of extraction of polyphenols from almond okara by optimizing and refining the conventional SLE method by studying the effects of the major extraction parameters (time, solid-to-solvent ratio, solvent composition, and temperature) on the recovery of phenolic compounds). Firstly, the optimal time and solid-to-solvent ratio for achieving the highest yield of phenolic compounds were established. Following this, the impact of temperature and solvent (ethanol and water) on the quality of the extracts was assessed by measuring their antioxidant activity. Secondly, the content of triglycerides and alpha-tocopherol in okara was quantified to ensure its relatively complete valorization, using the almond kernel as a reference.

## 2. Materials and Methods

### 2.1. Chemicals and Reagents

Alpha-tocopherol (analytical standard), gallic acid (GA), Trolox, (+) catechin, L-ascorbic acid, 1,1-diphenyl-2-picrylhydrazyl (DPPH), ammonium molybdate, sodium nitrite, sodium phosphate, and sodium hydroxide were purchased from Sigma-Aldrich Co., St. Louis, MO, USA. Folin-Ciocalteu’s phenol reagent (CAS number 12111-13-6) was used for total polyphenol quantification; acetonitrile (ACN), methanol (MeOH), and heptane were used for the chromatographic analysis of TG; and sample dilutions were obtained from VWR (Fontenay-sous-Bois, France). MeOH and ACN were purchased in gradient-grade purity. Aluminum chloride was from Panreac Quimica—Barcelona, Spain. Ethanol used for extraction and solvents used for UHPLC (analytical grade) were purchased from Carlo Erba—Val de Reuil, France. Visible spectra measurements were determined using a UV-2450 spectrophotometer (Spectrostar Nano, BMG Labtech, Ortenberg, Germany). Carbon dioxide (99.7% purity) was provided by Air Liquide (Paris, France).

### 2.2. Sample Preparation: Production of Almond Okara

Spanish almonds, sourced from a local organic store, were used to prepare almond milk. The whole almond kernels were soaked overnight at 4 °C, then blanched at 90 °C for 3 min. After blanching, the almonds were blended with ultrapure water (Millipore Integral 5 Milli-Q, Merck™, Trosly-Breuil, France) in a 1:9 weight-to-volume ratio. The resulting mixture was filtered through a muslin cloth to obtain the almond milk [26]. The freshly produced okara was immediately used to determine the dry matter content and optimize the extraction parameters for the dosage of bioactive compounds.

### 2.3. Dry Matter Determination of Okara

The freshly obtained okara’s dry matter (DM) content was analyzed in a ventilated oven at 105 °C. A specific amount of sample was weighed and dried for 24 h or until it reached a constant weight (AOAC, 1995).

### 2.4. Extraction Procedures and Experimental Design

#### Extraction Kinetics: Effect of Extraction Time on Total Polyphenols’ Yield

This preliminary study aimed to determine an optimal extraction time to optimize other solid–liquid extraction parameters and facilitate a more detailed experimental design. To achieve this, the extraction kinetics for 180 min using a solid-to-solvent ratio of 1:30 g/mL was investigated under nine conditions. These conditions varied in solvents (water, 25% ethanol, and 50% ethanol) and temperatures (20 °C, 40 °C, and 60 °C). Samples of the extracts were collected at 5, 10, 15, 30, 60, 90, 120, 150, and 180 min, and total polyphenol yield YTP was determined (as described in Section 2.5 below) and expressed as mg of gallic acid equivalent (GAE) per 100 g of dry almond okara:

(1) $$Y_{TP}:\;\frac{mg\;(TP)}{100\;g\;dry\;okara\;}\;=\frac{C*V}{M}$$

where C is the concentration of total polyphenols of the extract (mg GAE/mL), V is the volume of the solvent (mL), and M is the dry weight of the sample (g).

**Modeling of the kinetics of solid–liquid extraction: Peleg model**

To describe the evolution over time of extraction yields, the Peleg model, with parameters K1 and K2, was applied since in previous studies this mathematical model provided a precise estimation of the kinetics of the solid–liquid extraction process [27,28].

The constant K1 is Peleg’s maximum extraction rate (under the studied conditions), and K2 is Peleg’s maximum extraction yield (under the studied conditions). The experimental data obtained in the kinetic studies under the 9 experimental conditions were used to estimate the corresponding values for the K1 and K2 coefficients. For that, the Peleg model was used to plot the total polyphenol yield (YTP), tannin yield (YT), and total flavonoid yield (YTF) as well as its total antioxidant capacity (TAC) in the extraction solvent (as described in Section 2.6 and Section 2.7 below). The equation is presented below (Equation (2)):

(2) $$Y(t)=\left(\frac{t}{\left(\frac{1}{K1}\right)+\left(\frac{1}{K2}\right)*t}\right)$$

where t is the extraction time and Y(t) is the yield at time t expressed in mg/100 g dry okara.

The values of K_1_ and K_2_ at each experimental condition were obtained by searching to minimize the deviation (NRMSD) between the experimental data and the Peleg model. The normalized root mean squared deviation (NRMSD) criteria were calculated for each condition (Equation (3)):

(3) $$NRMSD=\frac{RMSD}{exp\;max}=\frac{1}{n}\sum_{p=1}^{n}{(\exp p-mod\;p)}^{2}$$

where n is the number of experimental points composing a kinetic curve for each curve in the present study, corresponding to the different sampling times; exp_p_ is the experimental value at point p; mod_p_ is the model value at point p; and exp_max_ is the maximum within the n experimental values.

- *Effect of the okara-to-solvent ratio on the total polyphenol yield*

The impact of the solid-to-solvent ratio was studied for 90 min at 60 °C by varying the amount of okara in 30 mL of solvent with ratios of 1:10, 1:20, 1:30, 1:40, and 1:50. Total polyphenol yield was calculated as described in Section 2.5 below.

- *Effect of ethanol–water mixtures at different percentages as well as extraction temperatures on the phenolic compounds content and antiradical activity of the extracts*

The effect of 0%, 25%, 50%, and 75% of ethanol, in addition to several temperatures (20 °C, 30 °C, 40 °C, 50 °C, and 60 °C), were compared on the different parameters. The optimal ethanol proportion and extraction temperature were determined by comparing the total polyphenol, tannin, and flavonoid yields and the antioxidant activity.

- *Optimization of phenolic compound extraction from okara: experimental design*

After setting the extraction time, an experimental design was carried out. A Box-Wilson procedure, also known as a Central Composite Design (CDD), was used to determine the effect of the main process variables and their interactions on various process responses while minimizing the number of required experiments. Three process variables, having a strong potential influence on the extraction process and the recovery of phenolic compounds, were selected: extraction temperature (X1 in °C), solvent composition (X2 in % ethanol in the solvent), and solid-to-solvent ratio (X3 in g okara per mL of solvent). The process responses were studied on the total polyphenol yield (YTP in mg GAE/100 g dry okara), tannin yield (YT in mg GAE/100 g dry okara), total flavonoid yield (YTF in mg CE/100 g dry okara), and the total antiradical capacity of the extracts (TAC in mg AAE/100 g dry okara). A total of 17 experiments were conducted, including three replicates at the center point. The experimental design is presented in detail in Table S1.

Experimental data obtained from these 17 experiments were used to build the model for the above-described yields (Y_TP_, Y_T_, Y_TF_, and TAC), and were calculated according to Equation (4). These 4 yields were expressed as functions of the three studied parameters, namely temperature (X_1_), ethanol percentage in the solvent (X_2_), ratio okara/solvent (X_3_), and all of their interactions:

(4) $$Y=a_{0}+a_{1\;}X_{1}+a_{2}X_{2}+a_{3\;}X_{3}+a_{11\;}X_{1}^{2}+a_{22\;}X_{2}^{2}+a_{33\;}X_{3}^{2}\;+a_{12}X_{1}X_{2}+a_{13}X_{1}X_{3}+a_{23}X_{2}X_{3}+a_{123}X_{1}X_{2}X_{3}$$

The objective was to determine the values of the regression coefficients (a_0_, a_1_, a_2_, a_3_, a_11_, a_22_, a_33_, a_12_, a_13_, a_23_, and a_123_) for each of these 4 yields by minimizing the deviation between the experimental and model data. To characterize these deviations, normalized root mean squared deviation (NRMSD) criteria were selected (Equation (3)).

### 2.5. Determination of the Total Polyphenol and Tannin Yields in Aqueous and Ethanolic Extracts

The Wiesneth and Jürgenliemk microplate spectrophotometric method was used for total polyphenol and tannin quantification [29].

- *Determination of the total polyphenol yield (YTP)*

Briefly, 25 µL of the extracts (aqueous or ethanolic extracts) or gallic acid (GA), used as a positive control for the calibration curve, were added to each well. Gallic acid solutions in different ethanol proportions (0%, 25%, 50%, and 75%) were prepared at various concentrations (0–200 mg/L), and calibration curves were calculated. Then, 125 µL of a 1/10th dilution of Folin-Ciocalteu’s reagent (mixture of phosphotungstic acid (H_3_PW_12_O_40_) and phosphomolybdic acid (H_3_PMo_12_O_40_)) were then added and shaken without light for two minutes at 40 °C. Finally, 100 µL of a 4% solution of Na_2_CO_3_ anhydrous in water was added to each well. The plate was incubated for an additional 2 h at 40 °C and the absorbance was measured at 690 nm using a spectrophotometer (Spectrostar Nano, BMG LABTECH GmbH, Ortenberg, Germany). The total polyphenol yield in the extract was expressed in gallic acid equivalent (mg GAE /100 g dry okara) [30,31,32].

- *Determination of the tannin yield (YT) with hide powder method*

To determine the YT yield, 0.5 mL of the same extracts were added to 10 mg of hide powder (Sigma-Aldrich Chemie Gmbh, Steinheim, Germany) to eliminate tannins by precipitation. The mixtures were stirred and incubated without light for 60 min. A centrifugation was conducted at 3046× *g* (4350 rpm in a TX-150 rotor, Model Megar Star 600, VWR) at room temperature for 5 min. The supernatants were then subjected to the same protocol as that described for TP with Folin-Ciocalteau’s reagent. Tannin yield (Y_T_) was obtained by subtracting the total tannin-free polyphenol content from the total polyphenol content of the same extract and was expressed in mg GAE/100 g dry okara [29,33].

### 2.6. Determination of the Total Flavonoid Yield (Y_TF_) in Aqueous and Ethanolic Extracts

This method involved a reaction between the aluminum ions and flavonoids present in the extracts under basic conditions. First, 1.5 mL of the extract was added to a mixture of 450 μL of 5% NaNO_2_, 900 μL of 10% AlCl_3_-H_2_O, and 4 mL of 1 M NaOH. Then, the mixture was stirred and incubated for 5 min before each addition. The final volume was made up to 15 mL with ultrapure water, and the absorbance was measured at 510 nm. Catechin solutions in different ethanol proportions (0%, 25%, 50%, and 75%) were prepared at various concentrations (0–500 mg/L), and calibration curves were calculated. The results obtained were expressed as mg (+)-catechin equivalent (CE)/100 g dry okara [34,35,36].

### 2.7. Antioxidant Activity

- *Evaluation of total antioxidant activity by the phosphomolybdenum method*

The total antioxidant capacity of the extracts was evaluated according to the method described by Prieto et al. [37]. The basic principle is based on the reduction of Mo (VI) to Mo (V) by the plant extract possessing antioxidant compounds and the subsequent formation of a green phosphate/Mo (V) complex at acidic pH. First, 0.1 mL of the sample extract was mixed with 1 mL of reagent solution (0.6 M sulfuric acid, 28 mM sodium phosphate, and 4 mM ammonium molybdate). Then, the tubes were incubated in a water bath at 95 °C for 90 min. After the samples were cooled to room temperature, the absorbance of each sample was measured at 695 nm against a blank with a spectrophotometer (Spectrostar Nano, BMG LABTECH, Ortenberg, Germany). Ascorbic acid in different solvent concentrations was used as the reference standard and the calibration curves were calculated (0–400 mg/L). The antioxidant activity was expressed as ascorbic acid equivalent (mg AAE/100 g dry okara). A high absorbance value indicates a high antioxidant activity [38].

- *DPPH free radical-scavenging capacity*

The free radical-scavenging activities of the extracts were determined according to the method described by [39,40], with slight modifications. This method uses the stable 1,1-diphenyl-2-picryl-hydrazil radical (DPPH from Sigma-Aldrich).

In its radical form, DPPH has an absorption band at 515 nm, which disappears upon reduction by an antiradical compound. Briefly, 20 µL of aqueous or ethanolic extracts were mixed with 180 µL of daily prepared DPPH solution (100 µM prepared in 80% ethanol). After incubation in a dark place for 60 min at room temperature, the absorbance of the mixture was measured at 515 nm against ethanol as a blank using a spectrophotometer (Spectrostar Nano, BMG Labtech). All experiments were carried out in triplicate. Trolox in 80% ethanol was used as the reference standard and the calibration curve was calculated (50–400-µmol/L). The antioxidant capacity was expressed as µmol Trolox equivalent (TE)/100 g dry okara.

### 2.8. Alpha-Tocopherol Quantification

- *Preparation of the sample*

The method used was described by [41] and adapted with slight modifications. Briefly, 0.5 g of crushed almond or okara was weighed into a glass screw-cap test tube and then 0.2 g of L-ascorbic acid, 10 mL of methanol, and 3 mL of 50% KOH solution were added. The mixtures were left to saponify overnight (18 h) at room temperature. The saponified sample was then transferred to a separatory funnel and extracted vigorously 3 times with 10 mL of n-hexane for 2 min. The combined hexane partitions were washed in a separatory funnel 3 times with 20 mL of distilled water until the pH was neutral. Then, the combined hexane partitions were dried over anhydrous sodium sulfate. The hexane was evaporated with a SpeedVac vacuum concentrator (Thermo-Electron) to obtain the dried residues. They were then dissolved in 1 mL methanol, filtrated through a 0.45 μm filter, and transferred into 2 mL amber HPLC vials. The calibration curve plotted for the α-tocopherol standard was calculated over a concentration range of 10–100 μg/mL.

- *Instrumentation and Chromatographic Conditions*

Determination of the α-tocopherol content was carried out by UHPLC-UV-MS with an Acquity UPLC^®^ H-Class Waters^®^ system (Waters, Guyancourt, France) equipped with two independent pumps, an automatic injector, a controller, a diode array detector (PDA) and a quadrupole (QDA) mass spectrometer with ESI as the ionization source. The stationary phase was a Waters^®^ Acquity BEH C18 column (2.1 × 50 mm, 1.7 μm) at 30 °C and connected to a 0.2 μm in-line filter. The mobile phase was composed of 100% methanol with an isocratic elution and a flow rate of 0.3 mL/min. The injection volume was 2 μL. The total analysis time was 6 min per sample. The spectrophotometric detection for vitamin E determination was performed at λ = 290 nm.

### 2.9. Analysis of Triglycerides

- *Preparation of the sample*

Almonds and okara samples were evaluated for their composition of triglycerides. For that, 1 g of almond (100% DM) and 1 g of okara (35% DM) were mixed with 10 mL of heptane, vortexed, then placed in an ultrasonic bath for 30–45 min. The mix was vortexed again and then filtered through a PTFE filter. The obtained extract was then diluted with a dilution factor of 1/100 before analysis.

- *Chromatographic system and mass spectrometry*

The experiments were conducted using the Nexera UC supercritical fluid chromatography system from Shimadzu Corporation (Kyoto, Japan) connected to a simple quadrupole mass spectrometer (LCMS 2020) with atmospheric pressure chemical ionization (APCI-MS), also provided by Shimadzu Corporation. All chromatograms were recorded on LabSolutions LCMS version 5.120 from Shimadzu Corporation. The method of analysis conditions was established based on previously published work [42]. Five octadecyl-bonded silica columns, totaling 75 cm in length, were used in tandem, including four Kinetex C18 (150 × 4.6 mm, 2.6 µm superficially porous particles, Phenomenex, Le Pecq, France) and one Accucore C18 (150 × 4.6 mm, 2.6 µm superficially porous particles, from Thermo-Electron, les Ulis, France). The column oven temperature was set at 17 °C. Isocratic analyses were performed over 80 min, with 88% of scCO_2_ and 12% of co-solvent composed of ACN:MeOH 90:10 (*v*/*v*). The total flow rate was 1.6 mL/min, and the injection volume was 1 µL for all samples. The back-pressure regulator was set at 100 bars and heated at 60 °C to mitigate the impact of CO_2_ cold depressurization.

Detection was achieved with APCI-MS, with the following operating conditions: source temperature 350 °C, desorption line temperature 250 °C, heat block temperature 200 °C, internal voltage 4.5 kV, drying gas flow rate 10 mL/min, and nebulizing gas flow rate 1.5 mL/min. Positive ionization mode was used with a scan speed of 15,000 u/s to track [M+H+] triglyceride ions. Total ion chromatograms (TIC) with *m*/*z* ranging from 50 to 1000 were used, together with individual single ion monitoring (SIM) for the expected ions in the TG species (571, 573, 575, 577, 595, 597, 599, 601, 603, 834, 852, 854, 856, 858, 860, 862, 874, 876, 878, 880, 882, 886, 888, 890, 892, and 884). Multiple ion chromatograms (MIC) with *m*/*z* ranging from 800 to 1000 were extracted to obtain cleaner traces. Relative quantification was carried out as previously described [42].

### 2.10. Statistical Analysis

The kinetic constants of the Peleg model (K_1_ and K_2_) and the regression coefficients ai, aij, and aijk of Equation (4) were determined through non-linear regression using a parameter estimation method based on the Newton algorithm, searching to minimize the deviation between the experimental and model data using the NRMSD criteria. The results are given as mean ± standard deviation of 3 replicates.

The measurements were performed in triplicate. The results are presented as mean ± standard deviation. Analyses were performed using two-way ANOVA followed by a Tukey post hoc test for comparisons between the extracts. Statistical analyses were performed using GraphPad Prism 10.

## 3. Results

### 3.1. Extraction Kinetics: Effect of the Extraction Time on Total Polyphenol Yield

**Figure 1:**
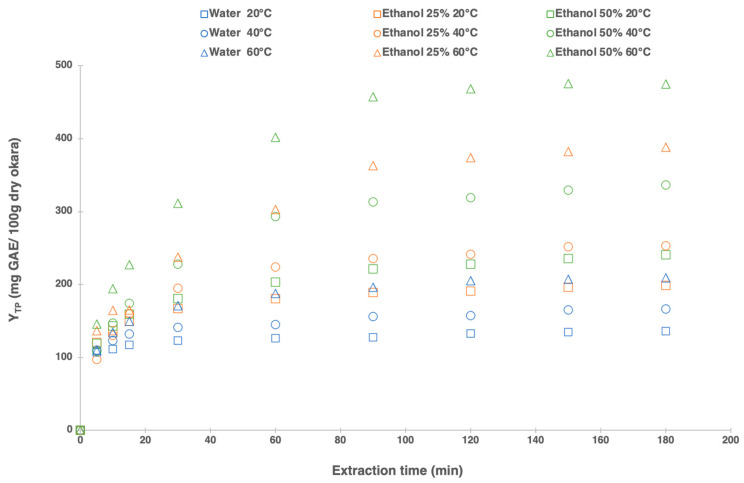
Solid/liquid extraction kinetics: effect of the extraction time on total polyphenols yield from okara with different temperatures (20 °C, 40 °C, and 60 °C) and ethanol concentrations (water, 25%, and 50% ethanol) for 180 min.

In addition to the effect of extraction time, two other parameters were studied simultaneously, temperature (20 °C, 40 °C, and 60 °C) and solvent composition (water, ethanol 25%, and ethanol 50%). Sample extracts were collected for 180 min. The results of the okara polyphenol extraction kinetics indicated that the polyphenol yield (Figure 1) gradually increased over time, reaching a relatively maximum level at 90 min, and then plateaued. At 90 min, the recovery of total polyphenol yield for water, 25% ethanol, and 50% ethanol at 60 °C reached 94% of the total polyphenol content (obtained at 180 min). Consequently, 90 min was opted as the standard extraction time for the subsequent optimization process. Numerous studies have shown that the solid–liquid extraction’ time is a crucial independent variable affecting the transfer of phenolic compounds. While longer extraction times generally improve efficiency and mass transfer, the increase in yield becomes negligible once solute equilibrium is achieved between the solid and the liquid phases [43,44].

Figure 1 also illustrates that both solvent mixture and extraction temperature significantly impact the extraction of polyphenols from okara. The total polyphenol yield varied from 127 mg GAE/100 g dry okara at 20 °C with water to 456 mg GAE/100 g dry okara at 60 °C with ethanol 50%. The effect of these two parameters was further examined in greater detail in this work (Section 3.4).

### 3.2. Modeling of Solid–Liquid Extraction Kinetic Parameters for TP Yield from Okara

The kinetic parameters of the Peleg model, K_1_ and K_2_, were determined for each of the nine conditions for the polyphenol extractions from okara. Analyzing these parameters can reveal the most influential factors and their interactions, offering valuable insights for optimizing the extraction process and maximizing polyphenol yields. The results presented in Table 1 indicated that the Peleg constant K_1_ decreased with increasing temperature and ethanol concentration, suggesting an inverse relationship between K_1_ and these two parameters.

Moreover, the K_1_ values for extracting total polyphenols from okara were higher when using water as the extraction solvent at 20 °C than when using ethanol. In contrast, the extraction capacity parameter K2 showed an opposite trend, increasing with higher ethanol concentration and higher temperatures. The optimal extraction capacity was achieved with 50% ethanol at 60 °C.

Additionally, the K_1_ values were higher when using 100% water at 20 °C compared to using ethanol as the extraction solvent. Conversely, the extraction capacity parameter K_2_ exhibited the opposite trend, increasing with higher ethanol concentrations and higher temperatures. The maximum extraction capacity was achieved with a 50% ethanol solution at 60 °C.

Even though water is known to be a good swelling agent that can efficiently diffuse through the plant matrix, making the extraction easier, other factors may impact the extraction rate. The higher K_1_ values observed with water might be due to the moisture state of the okara. Since undried okara was used, the extraction was already in progress, and contact with water facilitates the nearly instantaneous transfer of polyphenols. However, the results of K_1_ and K_2_ for 25% and 50% ethanol at higher extraction temperatures showed that the solvent’s polarity has a greater impact on the extraction rate. The 50:50 (*v*/*v*) water: ethanol mixture at 60 °C contributed to the higher extraction capacity observed, reaching up to 535 mg GAE/100 g okara.

Figure S1a–c shows the observed and predicted TP yields for all studied kinetics and for the nine extraction conditions. The low NRMSD values presented in Table 1 highlight the relatively good correlation between the experimental and predicted kinetic curves.

### 3.3. Solid-to-Solvent Ratio Effect on Total Polyphenol Yield

**Figure 2:**
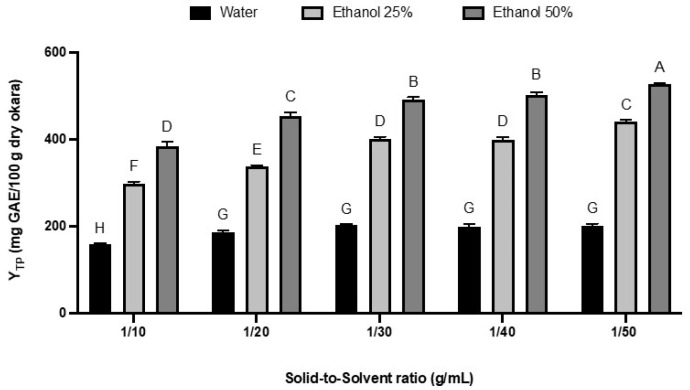
Effect of solid-to-solvent ratio on the total polyphenol yield expressed as mg GAE/100 g dry okara. The experiments were performed in triplicate. The amounts are shown as mean ± SD. A–H = differences between the extracts within a column (Tukey post-test, *p* < 0.05). Values not connected with the same letter are statistically different.

Two-way ANOVA tests show that both the solid-to-solvent ratio and the ethanol concentration in the solvent significantly affected the total polyphenol (TP) content (*p* < 0.0001). Although decreasing the solid-to-solvent ratio from 1:10 to 1:50 (*v*/*w*) positively impacted the TP yields, particularly with ethanol solvents, this effect was not directly proportional. The most substantial increase in TP yield occurred between 1:10 and 1:30 (*v*/*w*). Further reduction in the solid-to-solvent ratio resulted in only minor changes in TP yields (Figure 2).

It is well known that a lower ratio of solid-to-solvent is more beneficial for the extraction of phenolic compounds. This aligns with the principle of mass transfer, where the concentration gradient between the solid and the solvent drives the extraction process. Decreasing the solid-to-solvent ratio could amplify the concentration gradient and enhance this concentration gradient, which in turn improves the diffusion rate and makes the extraction more efficient [45]. Moreover, it was demonstrated that the amount of bioactive components extracted will not continue to increase once equilibrium is reached [46].

For the next steps, it was decided to maintain a solid-to-solvent ratio of 1:30 (*w*/*v*). This ratio provides a good balance between achieving a high total phenolic yield while maintaining it at high concentrations. Further reducing the ratio to 1:40 or 1:50 would slightly increase the yield but result in a less concentrated extract and higher costs associated with solvent removal.

### 3.4. Polyphenol Recovery as a Function of Temperature and Ethanol Percentage

**Figure 3:**
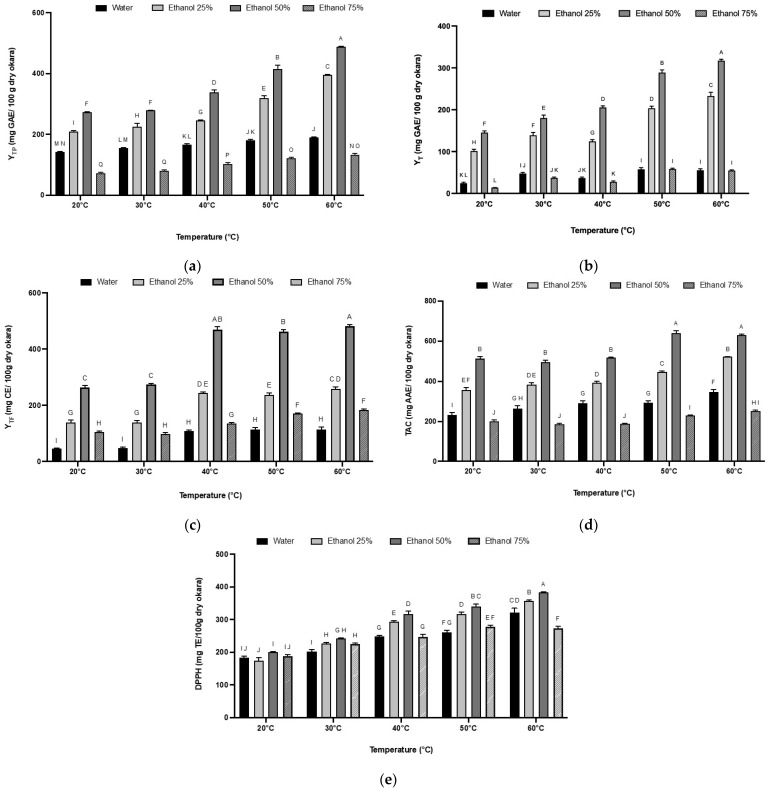
The effect of extraction temperature and solvent mixture on total polyphenol (**a**), tannin (**b**), and total flavonoid (**c**) yields, as well as antioxidant activity including total antioxidant capacity (**d**) and DPPH antioxidant activity Trolox equivalent (**e**). The experiments were performed in triplicate. The amounts are shown as mean ± SD. A–J: differences between the extracts within a column (Tukey post-test, *p* < 0.05). Values not connected with the same letter are statistically different.

The recovery of polyphenols as a function of temperature and ethanol percentage, after 90 min of extraction with a solid-to-solvent ratio of 1:30, showed that the extraction efficiency is significantly influenced by the temperature, with higher TP yields at 60 °C (Figure 3a). This effect of temperature can be attributed to several factors: the increased mass transfer and particle velocity, the enhanced solubility and diffusion coefficient of the solute in the solvent, and the decreased solvent viscosity and surface tension at higher temperatures, all of which accelerate mass transfer in a plant extract [47,48]. Furthermore, the high solvent temperature may promote the dissolution of cell wall polysaccharides into the solvent and weaken the integrity of the cell wall, thereby allowing phenolic compounds to pass easily into the solvent [49].

Additionally, the extraction was significantly improved by increasing the percentage of ethanol in the solvent mixture up to an ethanol percentage of 50%. While water is an efficient solvent for extracting polar molecules, greater extraction efficiency and a higher yields of phenolic compounds can be achieved by using organic or binary solvents for less polar compounds, [50]. For example, at 60 °C, the TP yield in 50% ethanol (489 mg GAE/100 g DM okara) was clearly higher than that of water extraction (190 mg GAE/100 g DM okara). This difference may be due to the high diversity and polarity of polyphenols found in okara; some are water-soluble (proanthocyanidins, flavonoid glycosides, etc.), while others are more ethanol-soluble (flavonoid aglycones, phenolic acids, etc.). Additionally, ethanol is well known to increase cell permeability by affecting the phospholipid bilayer, which in turn promotes solvent penetration and compound extraction [51].

However, polyphenol yields drastically decreased with 75% ethanol. Many studies have concluded that moderate concentrations of alcohol-like solvents like methanol and ethanol are more effective than higher concentrations. This difference could be due to the coagulation of proteins from the cell wall, which can result in diffusion resistance when solvents are added at higher concentrations [52,53]. Another reason could be the limited solubility of some hydrophilic phenolic compounds in ethanol.

### 3.5. The Effect of Extraction Temperature and Solvent Mixture on Tannin and Flavonoid Contents

The yields of tannin (Figure 3b) and flavonoids (Figure 3c) from the solid–liquid extraction of okara according to different factors were also determined. It is well established that phenolic compounds’ solubility depends on the plant matrix’s chemical nature and the solvent’s polarity [54].

Total tannins extracted from okara increased with increasing proportions of ethanol until maximum extraction was reached with 50% ethanol. The tannin content then decreased with 75% ethanol. Moreover, temperature elevation also has a positive effect on the tannin content. Tannin’s extraction efficiency was improved six-fold with 50% ethanol at 60 °C compared to water extraction at the same temperature and doubled compared to extraction at 20 °C, with 319, 55, and 145 mg GAE/100 g DM okara, respectively. These observations were consistent with several studies on total tannin extraction using organic solvents like ethanol and acetone. Although tannins are highly soluble in hot water, water has been proven to be an ineffective solvent for their extraction. This ineffectiveness may be due to the formation of tannin–protein complexes and the hydrogen bonds formed between the hydroxyl groups of the condensed tannins, which are major phenolic compounds in almond kernels, and water molecules, leading to colloid formation [55,56]. However, the effectiveness of ethanol could be attributed to its polarity, which allows for strong interactions with polar substances such as tannins [47,57].

The decrease in tannin yield with 75% ethanol could be due to the polymer number of the condensed tannins extracted with 75% ethanol. The study by Downey and Hanlin [58] highlighted that the total tannin extracted from Shiraz grape skin increased with increasing proportions of ethanol until a maximum extraction was reached at 50% ethanol in water, then declined in mixtures higher than 50% ethanol.

Flavonoids have demonstrated efficient extraction from plant matrices using ethanol as a solvent, as evidenced by studies such as those conducted by Chanda et al., and Rodriquez De Luna et al. [59,60]. The findings presented in Figure 3c align closely with these earlier investigations, notably, the gradual increase of flavonoid yields at temperatures exceeding 30 °C. Moreover, the yields were approximately doubled when using 50% ethanol at 40 °C, 50 °C and 60 °C, with 466 mg CE/100 g DM okara. This could be due to the previously stated arguments that higher temperatures weaken the matrix cell walls allowing a higher diffusion of phenolic compounds into the solvent. This increase in temperature allows a better liberation of bioactive compounds from plant cells by the decrease of solvent viscosity and the increase of molecular movement [61,62]. Furthermore, a higher ethanol percentage affects the permeability of the phospholipid layer, which could explain the higher flavonoid yield with the increase of ethanol to 50%, since flavonoids are localized in plants in the membrane layer between the lipid bilayer and aqueous phase. Consistent with the total polyphenol extraction, the recovery of tannin and flavonoid from okara was also enhanced by temperature and ethanol addition.

### 3.6. The Effect of Extraction Temperature and Solvent Mixture on the Antioxidant Activity of Okara Extracts

The antioxidant activity of okara extracts was determined using two bioassays. First, the phosphomolybdenum assay evaluated the total antioxidant capacity of the okara extracts with results expressed as ascorbic acid equivalent (AAE). The okara extract obtained at 60 °C with 50% ethanol showed the highest total antioxidant capacity, reaching 630 mg AAE/100 g DM of okara (Figure 3d). This extract also exhibited the highest polyphenol content (489 mg GAE/100 g DM okara). These values are in agreement with the TAC of almond kernels [63]. However, the values were not significantly different from those obtained at 50 °C (*p* > 0.05). This implies that the extracts at 60 °C and 50 °C have a strong antioxidant capacity compared to those obtained at lower temperatures and with different ethanol concentrations.

As for the free radical-scavenging capacity (DPPH assay), the extraction temperature had a more important effect on the antiradical activity than the solvent concentration. The highest values of 383, 357, and 322 mg TE/100 g DM okara were obtained with 0%, 25%, and 50% ethanol, respectively, at 60 °C (Figure 3e). These results showed that increasing temperature enhances the antioxidant activity in this case. This improvement could be due to the solvent’s low viscosity and enhanced mass transfer, which improve the extraction of phenolic compounds with greater bioactivity. Moreover, it is crucial to remember that the total activity of plant extracts may result from a combination of substances with antagonistic, additive, or synergistic action. This could be due to the previously described synergistic effect between some phenolic compounds and α-tocopherol [64].

### 3.7. Experimental Design

**Figure 4:**
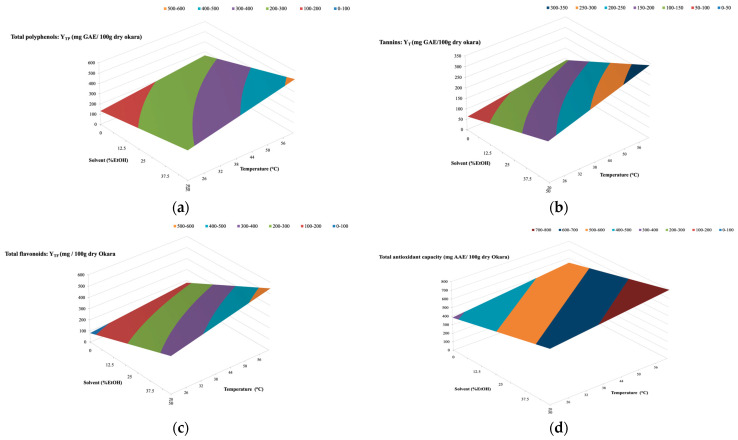
Response surface plots showing the interaction between ethanol concentration (X2) and extraction temperature (X1) at a fixed solid-to-liquid ratio 1:50 on Y_TP_ (**a**), Y_T_ (**b**), Y_TF_ (**c**), and TAC of okara extracts (**d**).

A Box-Wilson procedure was conducted to determine the adequate temperature and ethanol concentration as well as the solid-to-solvent ratio for optimizing polyphenols, flavonoids, and tannins yields, as well as the total antioxidant activity of extracts from okara. The previously observed positive effects of temperature, ratio, and ethanol concentration on the four studied yields were confirmed. An illustration is given in Figure 4, where the effects of ethanol concentration and temperature on TP, T, TF, and TAC yields are represented by three-dimensional graphs at a constant solid-to-solvent ratio (1:50 *w*/*v*) and time (90 min). While there were some differences in the amplitudes of these effects on the four studied yields, it was clear that overall, the increase in temperature and ethanol concentration in the solvent (up to 50%) led to an increase in all studied yields. The optimal multiple responses were obtained with an ethanol percentage of 50%, a temperature of 60 °C, and a solid-to-solvent ratio of 1:50, giving the following values for total phenolic yield (YTP) of 523 mg GAE, a tannin yield (YT) of 340 mg GAE, a total flavonoid yield (YTF) of 548 mg CE, and a total antioxidant capacity (TAC) of 779 mg AAE per 100 g of dry okara (Table 2). As mentioned previously, decreasing the ratio can increase the extraction yield due to the enhanced mass transfer.

The average NRMSD values (calculated on the basis of all 17 kinetics) were about 2.9% for YTP, 6.2% for YT, and 2.6% for both YTF and TAC, demonstrating a good correlation between the experimental and predicted data.

### 3.8. Alpha-Tocopherol Quantification

**Figure 5:**
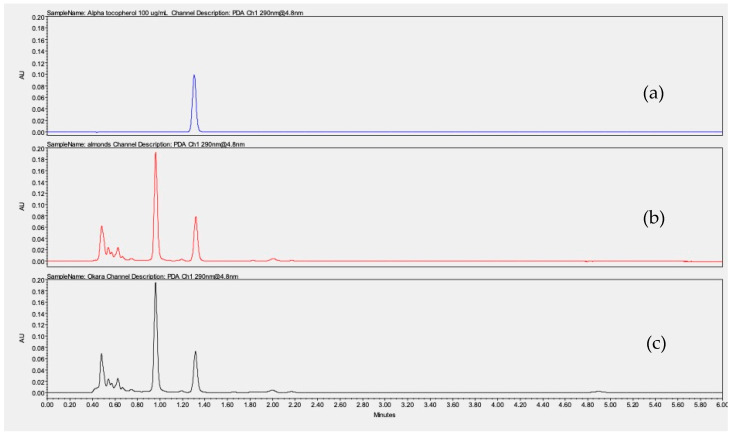
Chromatograms obtained by UHPLC-UV at 290 nm for alpha-tocopherol at 100 µg/mL (**a**), almond hexanic extract after saponification (**b**), and okara hexanic extract after saponification (**c**).

Vitamin E was quantified in almonds and okara by UHPLC-UV at 290 nm, yielding respective values of 15,600 µg and 14,400 µg per 100 g of dry sample, based on an alpha-tocopherol calibration curve. Figure 5 shows the chromatograms of an alpha-tocopherol solution at 100 µg/mL in methanol and almond and okara hexanic extracts after saponification (Figure 5). These results underlined that okara retains 92% of the vitamin E present in the almond. The vitamin E content in our almonds was consistent with the literature data, which reported values ranging between 5374 and 26,100 µg/100 g [53] depending on the origin of the almonds and the growing conditions [14,41]. These findings further support the potential of okara, especially since vitamin E is widely used in the cosmetic industry for its moisturizing and anti-aging properties.

### 3.9. Analysis of Triglycerides

**Figure 6:**
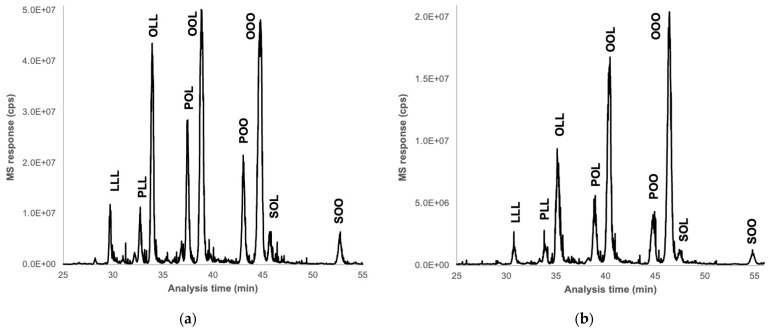
UHE/LPSFC-APCI-MS multiple ion chromatograms (MIC, from 800 to 1000 *m*/*z*) of almond (**a**) and okara (**b**). O represents oleic acid, L represents linoleic acid, P represents palmitic acid, and S represents stearic acid.

The TG content of almonds and okara showed very similar profiles. They mainly consist of OOL, OOO, and OLL triglycerides, where O represents oleic acid and L represents linoleic acid. These are followed by POL and POO, where P represents palmitic acid, and minor TG such as LLL, PLL, SOL, and SOO, where S represents stearic acid (Figure 6). These results are consistent with the literature [65,66]. Indeed, oleic acid, a mono-unsaturated omega-9 fatty acid, is the most abundant (70–80%), and linoleic acid (15%), a polyunsaturated omega-6 fatty acid, represents the primary lipids of interest in the almonds.

Additionally, since the okara sample was at 35% dry matter while the almond sample was completely dry, the relative quantification of TG in okara was equivalent to that in the almond. These findings suggest that the processing of almond milk does not significantly affect the lipid content of the resulting by-product, okara. This can be explained by the fact that the stages of milk processing are mainly aqueous extractions, which minimally impact the lipid content.

## 4. Conclusions

This work aimed to explore the valorization of okara as a potentially valuable byproduct by examining the effects of various solid–liquid extraction parameters (extraction time, solid-to-liquid ratio, ethanol concentration, and temperature) on the recovery of antioxidant phenolic compounds. The Peleg model described the kinetics of polyphenols extraction at different operating conditions well. The optimal extraction conditions were found to be a solid-to-solvent ratio of 1:50 *v*/*w*, 50% ethanol as solvent, a temperature of 60 °C, and an extraction time of 90 min. Under these conditions, the total polyphenol yield (YTP) was 523 mg GAE, the tannin yield (YT) was 340 mg GAE, the total flavonoid yield (YTF) was 548 mg CE, and the total antioxidant capacity was 779 mg AAE per 100 g of dry okara. Additionally, this study quantified the triglyceride and vitamin E levels in okara, which were found to be comparable to those in almonds. These findings demonstrated that, by optimizing the above parameters, the extraction of polyphenols from almond okara with the conventional SLE method can be maximized, making this process more efficient and the okara more valuable for functional food, nutraceutical, cosmetic, and pharmaceutical applications.

## Figures and Tables

**Table 1:** The kinetic parameters of the Peleg model, K_1_ and K_2,_ calculated for the nine extractions conducted with the three solvents at three temperatures.

	20 °C			40 °C			60 °C		
	Water	EtOH 25%	EtOH 50%	Water	EtOH 25%	EtOH 50%	Water	EtOH 25%	EtOH 50%
**K_1_**	90.97	50.15	36.49	58.33	27.25	24.32	37.79	22.70	29.00
**K_2_**	132.29	196.73	237.92	161.02	263.06	361.30	212.05	424.29	534.94
**NMRSD%**	2.5	2.8	3.9	3.5	1.5	5.6	2.8	2.4	2.7

**Table 2:** Experimental and model yields of total phenolic Y_TP_ (mg GAE/100 g dry okara), tannin Y_T_ (mg GAE/100 g dry okara), total flavonoid Y_TF_ (mg CE/100 g dry okara), and total antioxidant capacity from okara extracts (mg AAE/100 g dry okara).

Run	Y_TP_ (exp)	Y_TP_ (Model)	Y_T_ (exp)	Y_T_ (Model)	Y_TF_ (exp)	Y_TF_ (Model)	TAC (exp)	TAC (Model)
**1**	108.9	105.5	66.9	43.7	66.5	68.2	163.3	150.8
**2**	172.9	168.7	128.9	121.7	115.7	117.1	230.6	237.5
**3**	205.6	192.4	166.9	166.2	272.6	271.6	246.4	263.6
**4**	356.7	362	311.2	318.2	340.3	338.9	304.6	350.3
**5**	138.7	131.4	54.9	65.8	81.9	80.7	396.4	383.3
**6**	251.7	262.8	131.5	143.8	180.1	178.6	540.6	546.9
**7**	283.2	285.3	189.6	188.3	335	331.1	618.6	635.1
**8**	521.7	523	331.9	340.3	552.7	548.5	789.4	789.7
**9**	255.6	253.9	178.7	173.5	262.2	241.8	431.4	420.8
**10**	253.7	253.9	172.6	173.5	262.6	241.8	436.2	420.8
**11**	255.1	253.9	180	173.5	255.3	241.8	430.8	420.8
**12**	177.5	178.7	111.4	116	169.3	187.9	341.3	358.2
**13**	363.1	329.1	260.9	231	275.8	295.8	490.7	483.4
**14**	152.9	167.1	58.6	93.7	122.7	111.2	323.2	329.6
**15**	325.9	340.7	238.9	253.3	373.1	372.5	570.7	511.9
**16**	188.4	207.2	144.9	162.4	185.4	198.9	232.4	250.6
**17**	304.6	300.6	221.5	184.6	259.9	248.7	570.5	591
**NMRSD %**		0.029		0.062		0.026		0.026

## Data Availability

The original contributions presented in the study are included in the article/Supplementary Materials, further inquiries can be directed to the corresponding author.

## Supplementary Materials

The following supporting information can be downloaded at: https://www.mdpi.com/article/10.3390/foods13172828/s1, Table S1: The experimental points of each variable for the solid-liquid extraction of polyphenols from okara; Figure S1: Experimental and predicted total phenolic content during solid-liquid extraction from okara with three different solvents: water (a), ethanol 25% (b), and ethanol 50% (c) at three extraction temperatures (20 °C, 40 °C and 60 °C).
