# Supplementary material for: Optimizing the Extraction of Bioactive Compounds (Polyphenols, Lipids, and Alpha-Tocopherol) from Almond Okara to Unlock Its Potential as Functional Food
Source: Foods. 2024 Sep 5;13(17):2828. doi: 10.3390/foods13172828 (PMC11394736; doi:10.3390/foods13172828)
Supplement: Supplementary file 1 [file foods-13-02828-s001.zip › foods-3157709-supplementary.pdf]

foods

Supplementary Materials

Optimizing the Extraction of Bioactive Compounds (Polyphenols, Lipids, and Alpha-Tocopherol) from Almond Okara to Unlock its Potential as Functional Food

Table S1. The experimental points of each variable for the solid–liquid extraction of polyphenols from okara.

Run	Temperature °C (Codded Level)	Ethanol Concentration % (Codded Level)	Ratio Okara/Solvent g: mL (Codded Level)
**1**	20 (−1)	0 (−1)	1 :10 (−1)
**2**	60 (+1)	0 (−1)	1 :10 (−1)
**3**	20 (−1)	50 (+1)	1 :10 (−1)
**4**	60 (+1)	50 (+1)	1 :10 (−1)
**5**	20 (−1)	0 (−1)	1 :50 (+1)
**6**	60 (+1)	0% (−1)	1 :50 (+1)
**7**	20 (−1)	50 (+1)	1 :50 (+1)
**8**	60 (+1)	50 (+1)	1 :50 (+1)
**9**	40 (0)	25 (0)	1 :30 (0)
**10**	40 (0)	25 (0)	1 :30 (0)
**11**	40 (0)	25 (0)	1 : 30 (0)
**12**	20 (−1)	25 (0)	1 :30 (0)
**13**	60 (+1)	25 (0)	1 : 30 (0)
**14**	40 (0)	0 (−1)	1 : 30 (0)
**15**	40 (0)	50 (+1)	1 :30 (0)
**16**	40 (0)	25 (0)	1 :10 (−1)
**17**	40 (0)	25 (0)	1 : 50 (+1)

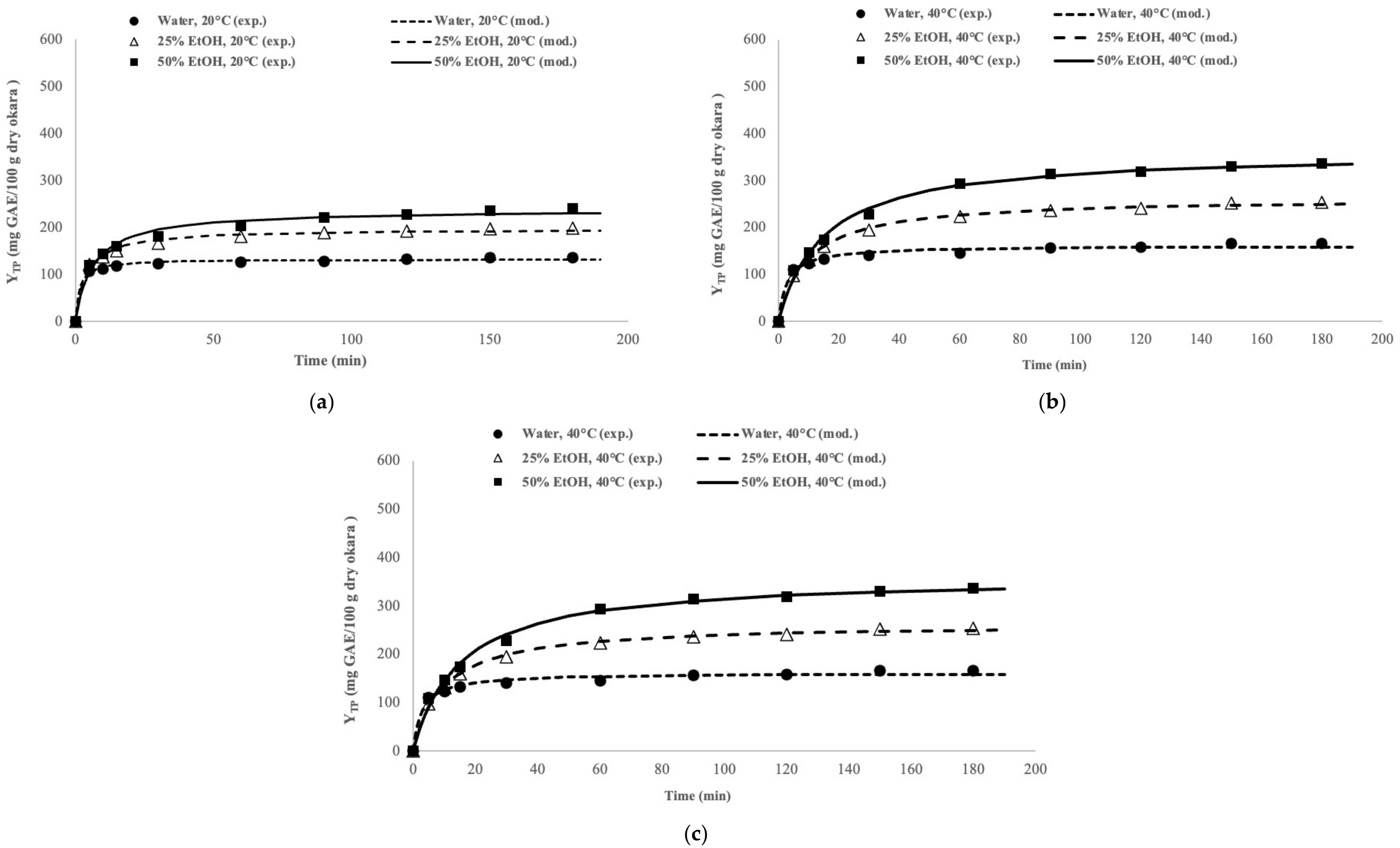

Figure S1. Experimental and predicted total phenolic content during solid–liquid extraction from okara with three different solvents: water (**a**), ethanol 25% (**b**), and ethanol 50% (**c**) at three extraction temperatures (20 °C, 40 °C and 60 °C).
